# Muscles of the male and female copulatory organs of *Bursaphelenchus mucronatus* and *Chiloplacus* sp. (Nematoda: Rhabditida)

**DOI:** 10.21307/jofnem-2021-107

**Published:** 2022-01-04

**Authors:** Alexander Ryss, Anatoly A. Petrov

**Affiliations:** 1 Zoological Institute of the Russian Academy of Sciences, Universitetskaya Naberezhnaya 1, St Petersburg, 199034, Russia

**Keywords:** Confocal microscopy, Copulative structures, Evolution, Morphology

## Abstract

Male and female copulatory organs figure prominently in nematode taxonomy, but the associated musculature remains insufficiently explored. The aim of this study was therefore to further our knowledge of the musculature of the vulva and male copulatory organs in nematodes by using phalloidin staining and confocal microscopy to examine two nematode species, *Bursaphelenchus mucronatus* and *Chiloplacus* sp. The musculature of the round vulva in *Chiloplacus* sp. comprises three pairs of radial vulval dilators and another pair of dilators of the anterior inner vulval plate. This arrangement is similar to that of the Rhabditida, but in *Chiloplacus* the anterior pair appears to have been transformed into the vulval plate muscles. The musculature of the slit-like vulva in *B. mucronatus* includes dilators of the vulval lips and external vulval flap, constrictors of the vulval slit and posterior transverse muscle bands. The opposing pairs of vulval dilators show quadrilateral symmetry as observed in the Rhabditida, but the constrictors running along the rim of the vulva have no counterparts in other species. The musculature of the male copulatory organ in *Chiloplacus* sp. comprises two pairs of spicule protractors and retractors and three pairs of gubernacular muscles. In *B. mucronatus*, as in the other Aphelenchoididae, the gubernaculum is absent and there is one pair of spicule protractors and two pairs of muscles inserted on the saddle (angular bend) of the spicules. The arrangement of the spicule saddle muscles resembles those of the gubernaculum, which may indicate that in this family the gubernaculum has become fused to the spicules. The literature review of muscles of nematode copulatory organs are given in a table for 15 muscle groups; it can be used for phylogenetic reconstruction and classification of the order Rhabditida.

Nematodes are an important component of the detrital food web that recycles biogenic substances in the biosphere (Odum, 1983). Among the saproxylic nematodes, the leading role is played by bacterial feeders and entomophilic fungal and plant feeders. The latter group includes species of the genus *Bursaphelenchus* that comprises 130 valid species (based on the authors’ database; Ryss et al., 2005; Ryss and Subbotin, 2017). Species of the genus have a major significance as plant pathogens. *Bursaphelenchus xylophilus* is a causal agent of pine wilt disease (PWD) in East Asia and since the 1990s also in Southern Mediterranean Europe, where it has spread from Asia as an invasive species (Mota et al., 1999).

*B. cocophilus* (Cobb, 1919) Baujard, 1989 and its vector, the weevil *Rhynchophorus palmarum* (Linnaeus, 1758), are the pathogenic associates causing the “red ring disease” of *Cocos nucifera* L., *Elaeis guineensis* Jacquin and other palms in the Caribbean region (Corbett, 1959; Goodey, 1960; Griffith and Koshy, 1990; Harrison and Jones, 2003; Silva et al., 2016). Data collected by the first author indicate that the elm nematode *B. ulmophilus* (Ryss et al., 2015) plays a role of an opportunistic pathogen in dieback diseases of the elm *Ulmus* spp. in the parks of St. Petersburg (Russia). This elm wood nematode accelerates the progression of the Dutch Elm Disease (Ryss et al., 2015) by facilitating the spread of the fungi along the tree trunk in sapwood (Polyanina et al., 2016; Ryss, 2016).

The molecular phylogeny of *Bursaphelenchus* (Kanzaki et al., 2015; Ryss et al., 2015; Gu et al., 2016; Ryss and Subbotin, 2017) and morphology-based evidence are only partially congruent warranting the search for new phylogenetically informative characters. The most important diagnostic characters of the nematode species are the structures of the anterior and posterior body regions, lateral fields, and copulatory organs. These structures have been studied primarily by differential interference contrast (DIC) and SEM, but recently these methods have been increasingly supplemented by confocal microscopy, with its ability to generate accurate three-dimensional (3D) reconstructions from optical sections.

The confocal study of nematodes, however, has so far been focused only on a few taxonomic groups. The favorite object of research is the nematode *Caenorhabditis elegans*, a model animal in neuroscience and developmental biology (Lints and Hall, 2009a, 2009b). Some confocal studies have been conducted on *Meloidogyne* spp. (Calderón-Urrea et al., 2016) and unstained collection specimens of aquatic nematodes have been studied by autofluorescence methods, among them the nematodes of the families Tobrilidae (Zullini and Villa, 2006) and Cyatholaimidae (Semprucci and Burattini, 2015). Beyond these examples, very little confocal research has been published.

This study was conducted to compare the muscular systems of copulatory organs in two phylogenetically distant species of nematodes using phalloidin staining in conjunction with confocal microscopy, with the objectives to evaluate the possibility of using the arrangement of copulatory muscles at the anatomical level to identify phylogenetically and taxonomically informative diagnostic characters that may distinguish the families within the order Rhabditida.

Two species of saproxylic nematodes were studied: the specialized fungal and plant feeder *Bursaphelenchus mucronatus* Mamiya and Enda, 1979 and the bacterial feeder *Chiloplacus* sp.; both are species occupying decaying wood and bark, but are clearly distant from each other in the phylogenetic tree of the order Rhabditida (Megen et al., 2009).

*Bursaphelenchus mucronatus* infects *Pinus sylvestris* L. using *Monochamus* spp. (Cerambycidae) vectors; it combines mycophagy with parasitic feeding on plant cells in the resin canals, while *Chiloplacus* are commensals of longhorn beetle larvae inhabiting the bark of pines and feeding on bacteria. A further imperative for this study was to study the morphological adaptations of nematodes to the different modes of mating.

## Materials and methods

### Nematode samples and extraction

Two nematode species were studied: *Bursaphelenchus mucronatus* Mamiya and Enda, 1979 (European type) and *Chiloplacus* sp., both belonging to the class Secernentea: order Rhabditida according to the classification of De Ley and Blaxter (2002). The isolates of both species are maintained in the Live Nematode Collection at the Zoological Institute of the Russian Academy of Sciences, St. Petersburg.

*Bursaphelenchus mucronatus* (family Aphelenchoididae) is a fungal and plant feeder using *Pinus sylvestris* L. as a plant host. The nematodes were extracted from larval tunnels of the pine sawyer beetle *Monochamus galloprovincialis* (Olivier, 1795) in the wood of *Pinus sylvestris* (Tomsk region of Russia, Tomsk district, Timiryazevo settlement, forest farm “Timiryazevskoye”, GPS: 56°27.597’N 084°51.157’E). Wood was collected on 19 June 2015.

*Chiloplacus* sp. (family Cephalobidae) is a bacterial feeder and commensal of longhorn beetles. The nematodes were collected in July 2006 and extracted from larval galleries of *Monochamus galloprovincialis* (Cerambycidae) in the wood of *Pinus pinaster* (Aiton, 1789) (Portugal, Évora, Polo da Mitra, GPS: 38°31’44.7”N 8°01’00.6”W).

### Nematode cultivation

To obtain mass suspensions of nematode isolates, they were propagated on a standard culture of an asporogenous mutant strain of the fungus *Botryotinia fuckeliana* (de Bary) Whetzel, 1945 (the name of teleomorph stage) (syn. *Botrytis cinerea* Pers. 1794). The fungus was grown on sterile potato sucrose agar (PA) at 20–22°C. Five days after fungus inoculation, the agar surface was occupied by the mycelium lawn. A suspension of 50–100 nematode individuals was inoculated into fungus culture. In 10–14 days, the nematodes multiplied reaching abundance of 3,000–10,000 individuals at different life cycle stages and devastating the fungus lawn (Ryss, 2015).

*Chiloplacus* sp. is not a fungal feeder and according to its stoma structure the genus can be characterized as a bacterial feeder (Yeates et al., 1993; Wilson and Khakouli-Duarte, 2009). However, these nematodes multiplied in the *B. fuckeliana* culture feeding both on fungus and unidentified ectosymbiotic bacteria, which were present on the nematode surface coat and multiplied rapidly in PA media; these bacteria serve as food for their nematode hosts. The nematodes were capable to multiply on the PA medium without fungus while feeding on their ectosymbiotic bacteria; however, in the PA medium with *B. fuckeliana,* the population growth was faster and more stable.

The arrangement of mating pairs could be easily seen at the spots where agar was sufficiently thin and on the lids of Petri dishes.

### Fixation, staining and manufacture of collection slides

The nematodes were fixed with hot 4% formaldehyde in 0.01M PBS for 1 h and then transferred to 0.01 M PBS containing 0.05% (w/v) sodium azide for storage. Permanent preparations of nematodes were made using a modification of the Seinhorst method (Ryss, 2003, 2017).

For confocal microscopy, the nematodes were permeabilized for 2–3 hr with 0.25% Triton X-100 in 0.01 M PBS (Tr-PBS) and incubated in 20  µg/ml proteinase K in 0.01 M PBS for 5 hr. Permeabilized animals were rinsed 3 × 15 min in Tr-PBS, stained for 5–10 hr in Alexa Fluor 555 phalloidin (1:150) (Thermo Fisher Scientific), rinsed in 0.01 M PBS for 15  min and mounted on slides with Vectashield (Vector Laboratories Inc., Burlingame, California, USA). The optimal time of proteinase incubation was determined experimentally: a 5-hr incubation provided sufficient permeabilization of the cuticle to stain the whole body of the worm, but was not long enough to digest filamentous actin in the muscles.

In addition, freeze-cracking technique (Duerr, 2013) was used as an alternative to proteinase permeabilization. In freeze-cracking of nematodes, live animals were placed in a small droplet on a slide, compressed under a second slide placed perpendicular to the first, and immediately transferred into a refrigerator at –25°C. After 1 h, the slides were taken from the refrigerator and their ends were quickly pulled together to bring the slides in parallel position thus cracking the body cuticle. The animals on the lower slide were fixed with 4% formaldehyde in 0.01M PBS for 1 hr. The subsequent steps for phalloidin staining were performed as described above starting from permeabilization in Triton-PBS, but without proteinase incubation. While nematodes species were cultured, about 1000 specimens of each species were stained with phalloidin, from which the adult nematodes were selected for confocal microscopy observations (three and two specimens for vulval muscles in *B. mucronatus* and *Chiloplacus* sp., respectively, and six and eight specimens for male copulatory organs in *B. mucronatus* and *Chiloplacus* sp., respectively); other individuals were also checked to avoid occasional artifacts and contradictions in the results.

### Morphological study and reconstructions

Confocal images were collected using a Leica TCS SP5 confocal laser scanning microscope at the “Taxon” Research Resource Centre (Zoological Institute of the Russian Academy of Sciences, St. Petersburg, Russia; http://www.ckp-rf.ru/ckp/3038/?sphrase_id=8879024). Three-dimensional reconstructions of confocal Z-stacks were made by manual data segmentation and automatic volume rendering using Avizo 8.1 (FEI Visualization Sciences Group, Burlington, MA). Cuticular structures and their relationships with muscles were visualized in the reflection mode of the confocal microscope as described in Petrov et al. (2016).

Where muscle homologies were evident, the muscles were named and labeled according to their names for *C. elegans* as summarized in Wormatlas (Lints and Hall, 2009a, 2009b).

## Results

### Female copulatory organs

#### Bursaphelenchus mucronatus

**Figure 1:**
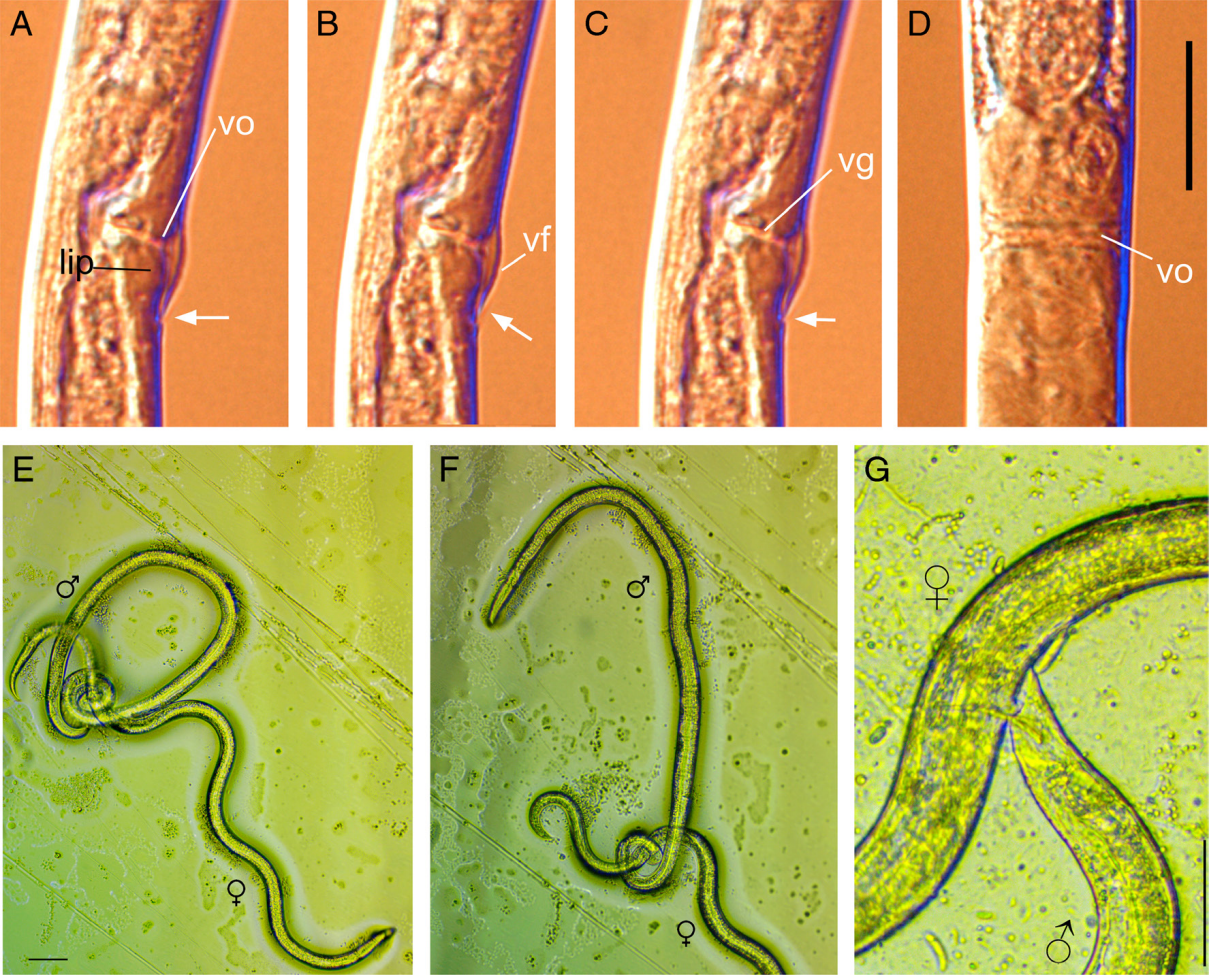
Vulval region of *Bursaphelenchus mucronatus* (A–D) and mating in *B. mucronatus* (E, F) and *Chiloplacus* sp. (G). (A–C) Series of lateral views of the vulval region at different optical levels. (B) Vulval flap looks flap-shaped with its ending not reaching the body wall (arrow). (A, C) Vulval flap is fold-shaped at the margins of the vulva (arrow): the fold continues to the body wall. Abbreviations: lip—posterior vulval lip; vf—vulval flap; vg—vagina; vo—vulval opening. (Scale bars: A–D = 30 µm; E–G = 50 µm).

**Figure 2:**
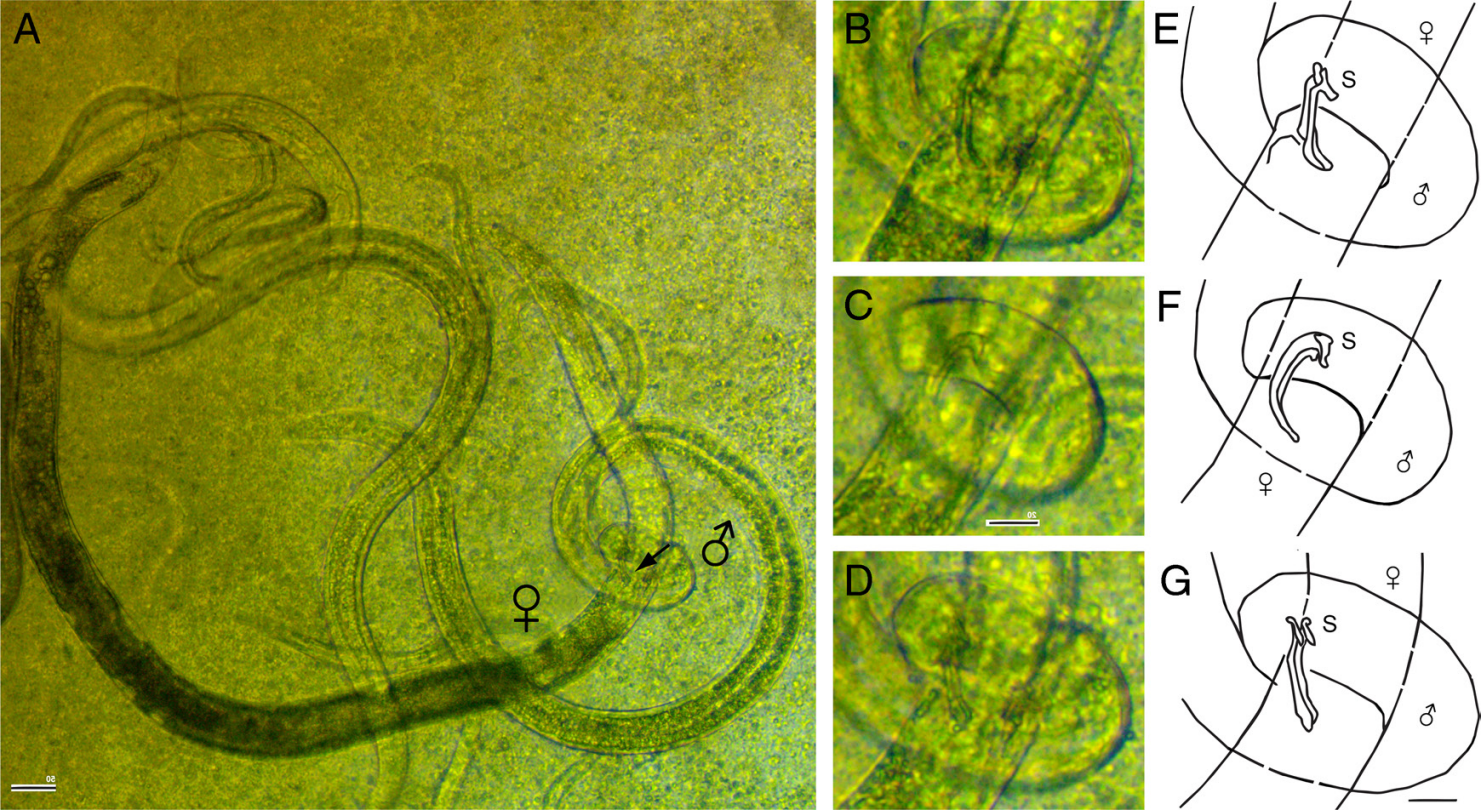
Mating of *Bursaphelenchus mucronatus* (from orig. video). (A) Arrangement of the male and female during mating. (B–D) Position of spicules inserted into the female during mating (enlarged from A). (E, F) Drawings highlighting details of B–D (Scale bars: A = 50 µm; B–G = 20 µm).

**Figure 3:**
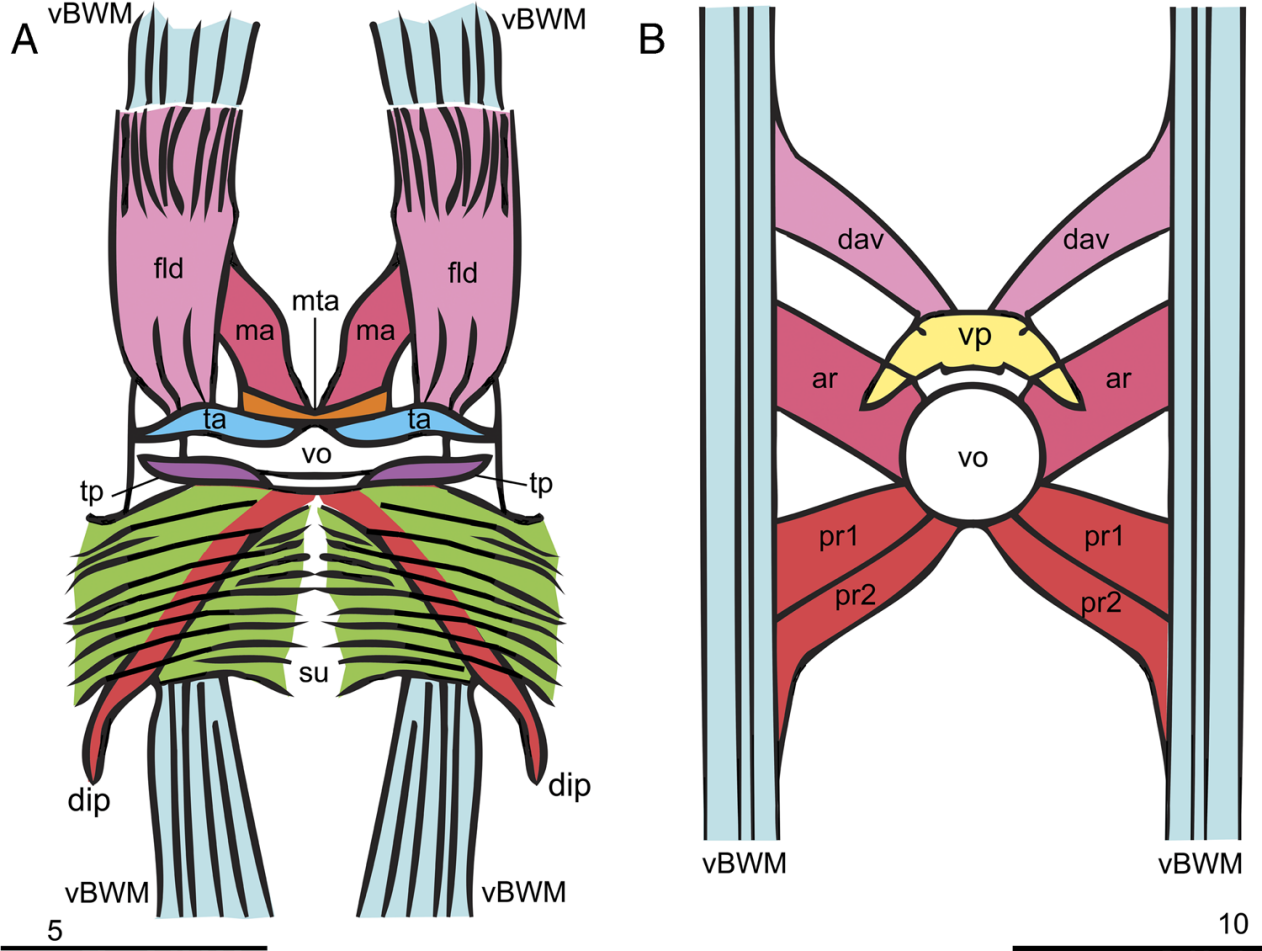
Schematic representation of the vulval muscles of *Bursaphelenchus mucronatus* (A) and *Chiloplacus* sp. (B). Abbreviations: ar—anterior radial vulval dilators; dav—dilator of anterior vulval lip; dip—dilator of posterior vulval lip; fld—dilator of vulval flap; ma—dilator of anterior vulval lip; mta—medial transverse constrictor of anterior vulval lip; pr (pr1, pr2)—posterior radial vulval dilators; ta—transverse constrictor of anterior vulval lip; tp—transverse constrictor of posterior vulvar lip; su—suspensor of posterior vulvar lip; vBWM—ventral body wall muscle; vo—vulval opening; vp—anterior cuticular vulval plate (Scale bars: A = 5 µm; B = 10 µm).

**Figure 4:**
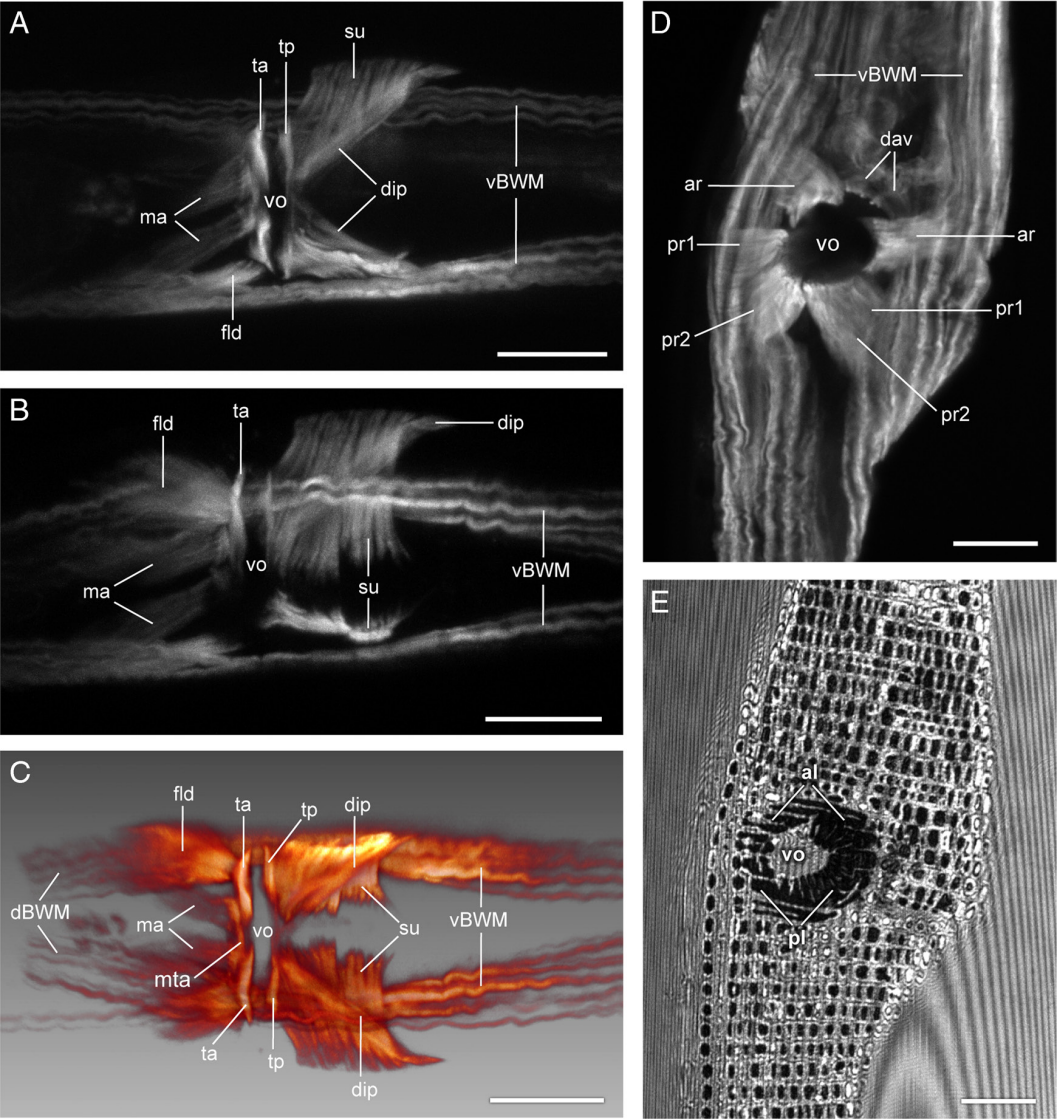
Vulval region of *B. mucronatus* (A–C) and *Chiloplacus* sp. (D, E). In (A–C) anterior is to the right, in (D, E) anterior is up. (A) Vulval musculature close to the dorsal side of the body. (B) Vulval musculature close to the ventral side of the body. (C) Three-dimensional reconstruction of the vulval musculature. (D) Vulval musculature close to the ventral side of the body. (E) Cuticle of the vulval region in reflected light. Abbreviations: al—anterior vulval lip; pl—posterior vulval lip; other abbreviations as in Figure 2 (All scale bars = 10 µm).

(Figs. 1A–F, 2, 3A, 4A–C).

*Vulva and vagina.* The vulva of the female is covered ventrally by a vulval flap, an arc-shaped cuticular fold that looks like a single anterior shield in lateral view (*vf,* Fig. 1A–C). The vulval flap consists of an anterior arch and lateral folds ending posterior to the transversal vulval slit (Fig. 1A–C).

The vulval musculature comprises: i) muscles of the vulval slit (constrictors), ii) muscles of the inner vulval slit and muscles of the external vulval flap (dilators), and iii) muscles of the posterior vulval lip (dilators and suspensors). During mating, the support for inserted spicules is provided by the muscles of the vulval flap and vulval lips (Figs. 1A–F; 2).

The constrictors of the vulval slit comprise two pairs of slender transverse muscles, one located along the anterior (*ta*) and the other along the posterior lip of the vulval slit (*tp*) (Figs. 3A; 4A–C). The longitudinal dilators of the anterior vulval lip (*ma*) are a pair of muscles attached medially to the anterior lip at the same spot, where the opposite anterior constrictors of the vulval slit (*ta*) meet at the body midline. Anteriorly, the *ta* muscles are bordered by a narrow unpaired transverse muscle (*mta,* Figs. 3A; 4C). The *ma* dilators continue anteriorly on either side of the ventral hypodermal chord to join the ventral body wall near the ventral body wall muscles (*vBWM*) (Figs. 3A; 4A–C).

The vulval flap dilators (*fld,* Figs. 3A; 4B, C) are a pair of broad longitudinal muscles that arise from the cuticular margins of the arc-like vulval flap and extend forward to attach to the *vBWM* muscles (Figs. 3A; 4B, C).

The posterior lip is associated with two pairs of muscles. A pair of wide transverse suspensors of the posterior lip (*su*) arises from the ventral hypodermal chord and extends dorsolaterally underneath the *vBWM* muscles to anchor on the body wall near the lateral hypodermal chords (Figs. 3A; 4A–C). A pair of spindle-shaped inner diagonal dilators (*dip*) originates on the body wall near the lateral hypodermal chords at the posterior edges of the inner suspensors (*su*) and runs beneath these muscles in the anteromedial direction to insert submedially on the posterior vulval lip, at about the same spot as the *tp* muscles (Figs. 3A; 4A–C).

#### Chiloplacus sp.

(Figs. 1G, 3B, 4D, E).

The vulval musculature of the *Chiloplacus* sp. females comprises three pairs of radial vulval dilators (*ar, pr1, pr2*) and one pair the anterior vulval lip dilators (*dav,* Figs. 3B; 4D). The radial dilators include one pair of anterior muscles (*ar*) and two pairs of posterior muscles (*pr1, pr2*). Each radial muscle originates on the body wall dorsal to the *vBWM* and inserts on the rim of the round vulvar opening. The dilators of the anterior lip (*dav*) are a single pair of muscles inserted on the arcuate cuticular plate of the anterior vulval lip (*vp*) (Figs. 3B; 4D). Each *dav* muscle runs anteriorly to end on the *vBWM* muscle of the corresponding body side (Figs. 3B; 4D).

### Male copulatory organs

#### Bursaphelenchus mucronatus

(Figs. 2, 5A–D, 7)

**Figure 5:**
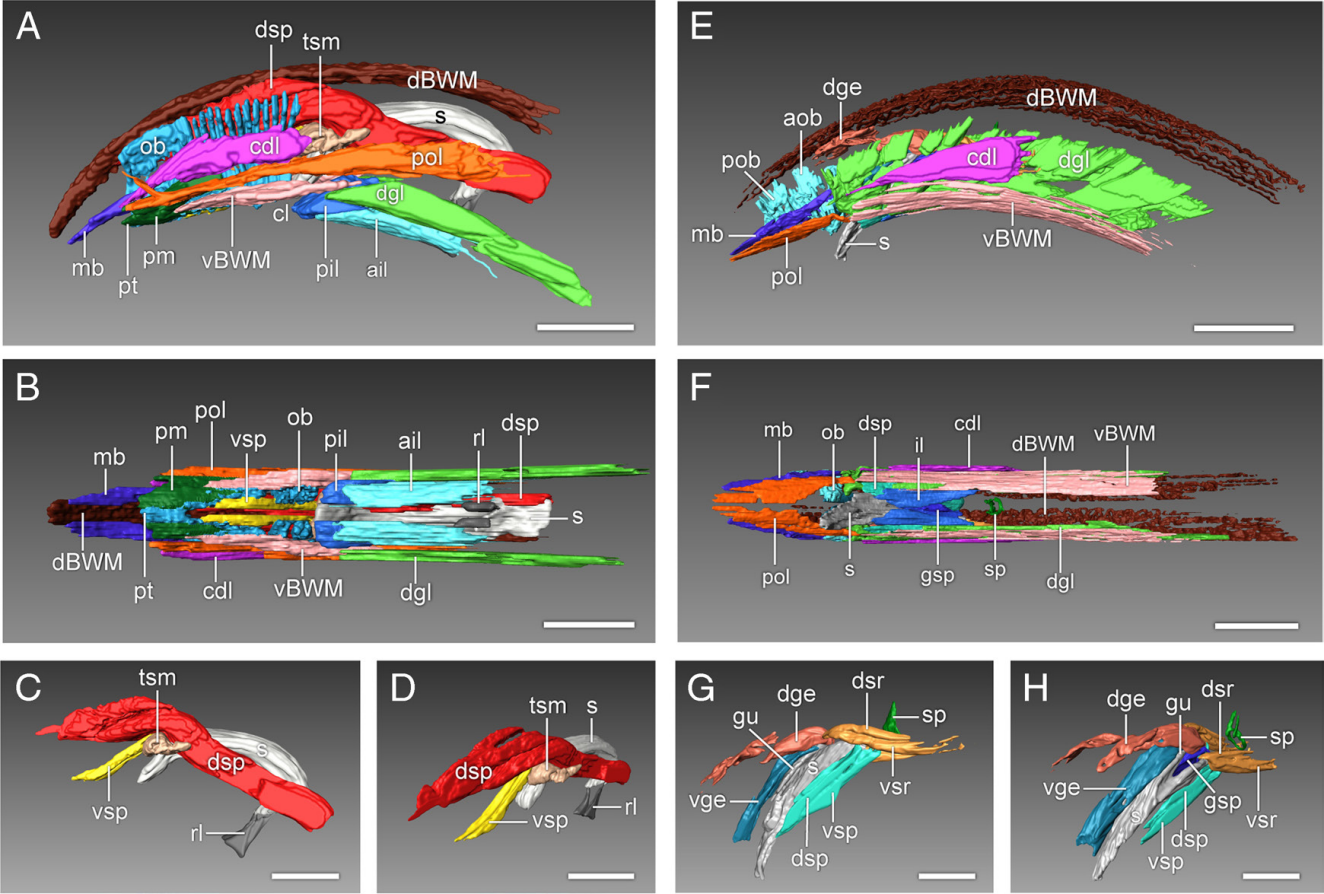
Three-dimensional reconstructions of the male caudal muscles of *Bursaphelenchus mucronatus* (A–D) and *Chiloplacus* sp. (E–H) generated using manual data segmentation in Avizo software. (A, E) Lateral view of external tail muscles. (B, F) Ventral view of external tail muscles. (C, G) Lateral view of spicular muscles. (D, H) Posterolateral view of spicular muscles. Abbreviations: ail—anterior inner longitudinal muscle; aob—anterior oblique muscle; cdl—caudal longitudinal muscle; cl—cloaca; dBWM—dorsal body wall muscle; dge—dorsal gubernaculum erector; dgl—diagonal muscle; dsp—dorsal spicule protractor; dsr—dorsal spicule retractor; gsp—gubernacular spicule protractor; gu—gubernaculum; mb—bursa muscle; ob—oblique muscle; pil—posterior longitudinal muscle; pm—papillary muscle; pob—posterior oblique muscle; pol—posterior outer longitudinal muscle; rl—rostral ligament; s—spicule; sp—sphincter muscle between mid-intestine and cloaca; tsm—transverse saddle muscle; vBMW—ventral body wall muscle; vge—ventral gubernacular erector; vsp—ventral spicule protractor; vsr—ventral spicule retractor (Scale bars: A–D, G–H = 10 µm; E, F = 20 µm).

**Figure 7:**
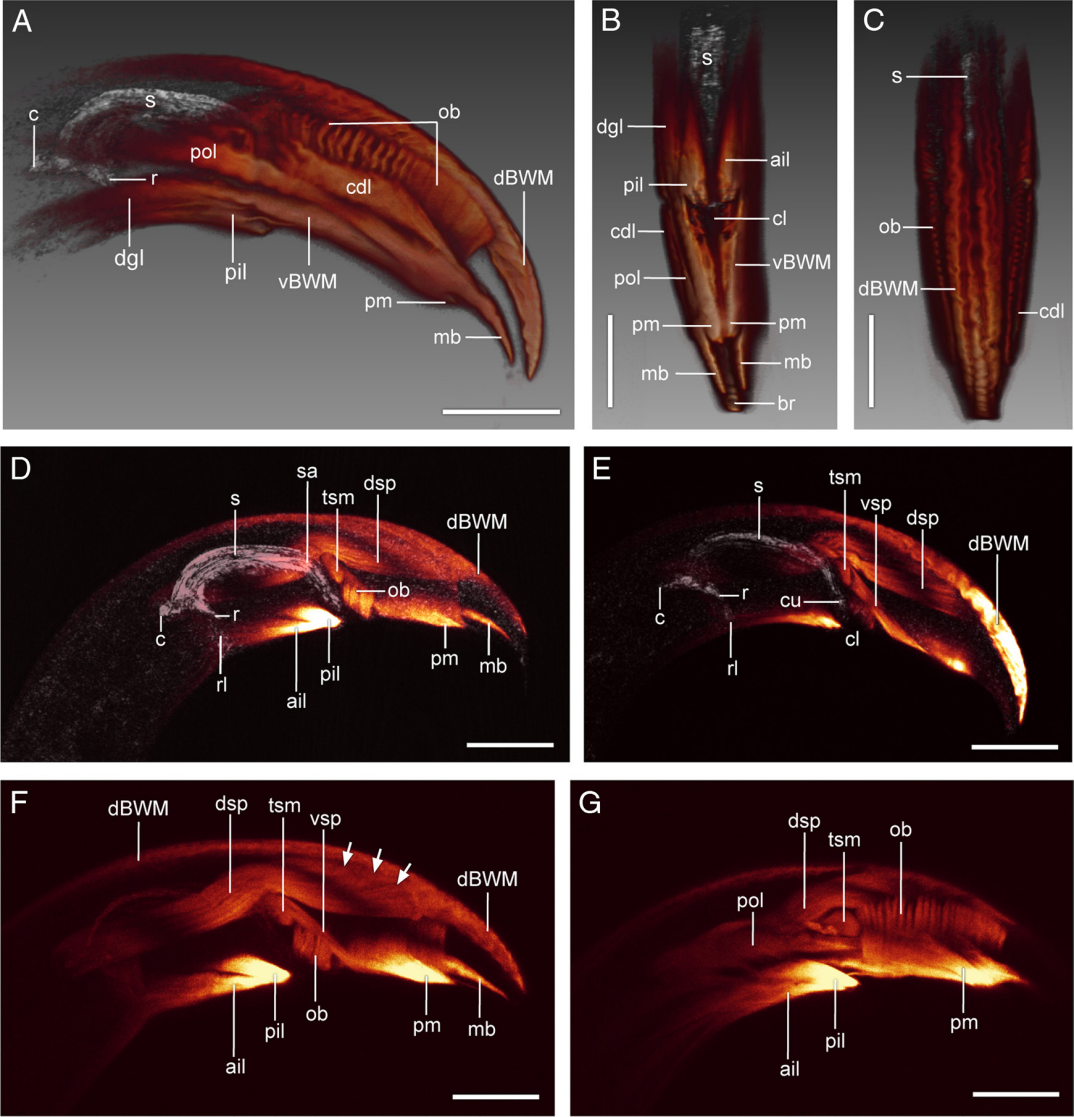
Three-dimensional reconstructions (A–C) and maximum projections (D–G) of the tail region of males of *B. mucronatus* showing musculature of the male copulatory organ and the bursa (orange) and spicule in reflected light (grey). (A, D–G) Lateral view. (B) Ventral view; (C) Dorsal view. Arrows (F) indicate muscle attachment points on the dorsal roof of the tail. Abbreviations: ail—anterior inner longitudinal muscle; br—bursa; c—condylus; cdl—caudal longitudinal muscle; cl—cloaca; cu—cucullus; dBWM—dorsal body wall muscle; dgl—diagonal muscle; dsp—dorsal spicule protractor; mb—bursa muscle; ob—oblique muscle; pil—posterior longitudinal muscle; pm—papillary muscle; pol—posterior outer longitudinal muscle; r—rostrum; rl—rostral ligament; s—spicule; sa—spicule saddle; tsm—transverse saddle muscle; vBMW—ventral body wall muscle; vsp—ventral spicule protractor (All scale bars = 10 µm).

1. Body wall muscles (*BWM: dBWM* and *vBWM*).

The *BWM* are the main muscles of the body; there is one pair of subdorsal bands (*dBWM*) located on each side of the dorsal hypodermal chord (Figs. 5A, B; 7A, C–F) and one pair of subventral bands (*vBWM*) situated between the ventral and lateral hypodermal chords on either side of the body (Figs. 5A, B; 7A). The *dBWM* muscles run posteriorly to the caudal tip of the body (Figs. 5A, B; 7A, C–F). The *vBWM* muscles continue to the caudal papillary tubercle (Figs. 5A, B; 7A, B).

2. Longitudinal muscles (*ail, pil, cdl, pol, pm*).

The inner longitudinal muscles, one anterior (*ail*) and one posterior (*pil*), extend posteroventrally from their origin on the subventral body wall to insert at the anterior rim of the cloaca. The *ail* lies dorsal to the *pil* and the muscles remain in tight contact with each other along most of their length (Figs. 5A, B; 7B, F, G). The paired caudal muscles (*cdl*) originate broadly at the ventrolateral sides of the body wall just anterior to the cloaca and continue posteriorly to the *mb* muscles (Figs. 5A, B; 7A–C). At the level of the cloaca, the *cdl* muscle forms a contact with the posterior longitudinal muscle (*pol*, Figs. 5A; 7 A). The posterior outer longitudinal muscle (*pol*) lies ventrolaterally between the *cdl* and *vBWM*. From its broad anterior attachment to the lateral body wall above the *dgl* muscles, it runs posteriorly toward the tip of the tail to terminate narrowly at the anterior end of the *mb* muscle (Figs. 5A, B; 7A, B, G). Near its posterior end the *pol* muscle gives off a narrow dorsal branch that ends on the lateral side of the tail (Fig. 5A). The papillary muscles (*pm*) arise internally from the ventral parts of the *ob* muscles on each side of the body and proceed posteriorly to attach to the body wall in the area of the papillary tubercle (Figs. 5A, B; 7A, B, D, F, G).

3. Precloacal copulatory diagonal muscles (*dgl*).

Diagonal muscles (*dgl*) are a pair of precloacal muscles lying ventral to the *pol* and superficial to the inner longitudinal muscles (*ail* and *pil*) (Figs. 5A, B; 7A, B). These muscles extend posteriorly from their attachment on the ventrolateral body wall and end submedially at the lateral borders of the cloaca.

4. Postcloacal copulatory oblique muscles (*ob*).

The oblique post-cloacal muscle is a broad muscle band stretching between the bursa and the cloaca to the inside of the *vBWM* and *cdl* muscles (Figs. 5A; 7A, C, D, F, G). Ventrally, the posterior part of the muscle is anchored on each side of the body midline and its anterior part is attached to the body wall lateral to the cloaca; dorsally, the muscle is attached to the dorsolateral body wall. The muscle is a single continuous sheet in its posterior third, but anteriorly is split into a series of 11–15 parallel myofibers (Figs. 5A; 7A, G).

5. Spicule and spicular muscles.

**Figure 6:**
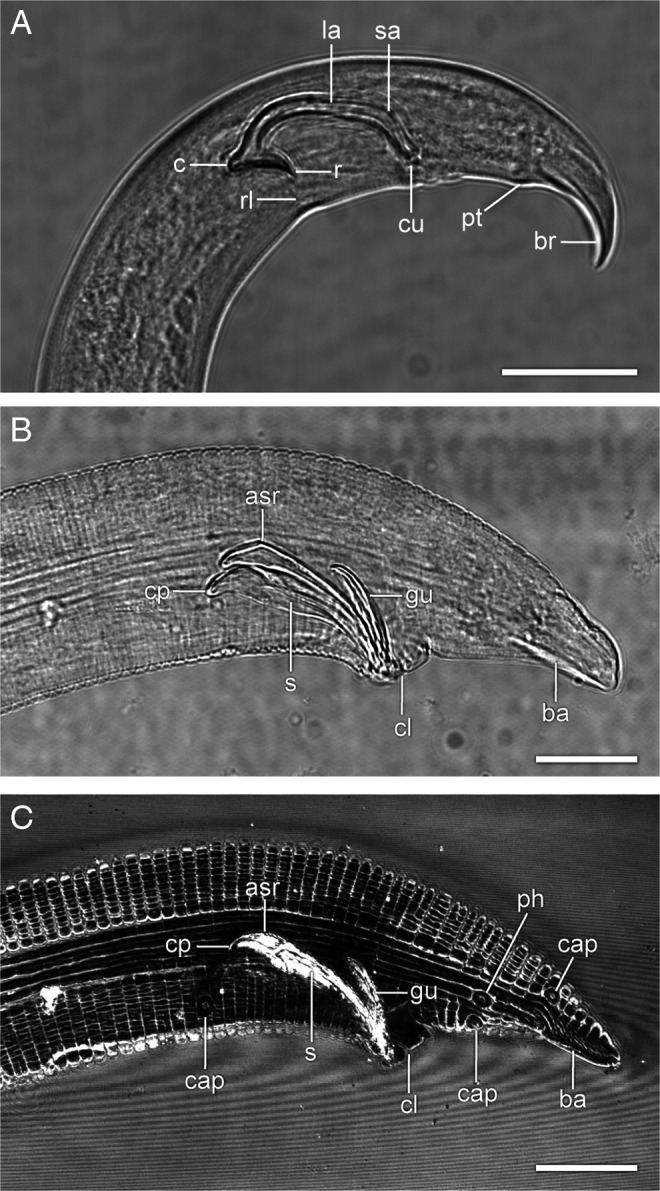
Tail region of males of *B. mucronatus* (A) and *Chiloplacus* sp. (B, C). (A, B) Bright field. (C) Reflected light. Abbreviations: asr—anterior spicule ridge; ba—bursal alae; br—bursa; cap—caudal papillae; c—condylus; cl—cloaca; cp—spicule capitulum; cu—cucullus of male spicule; gu—gubernaculum; la—lamina of male spicule; pt—papillary tubercle; s—spicule; sa—spicule saddle. (Scale bars = 20 µm).

Spicules are paired cuticular copulatory hook-like structures inside a Y-shaped proctodeum (cloacal sac). The spicule capitulum is flattened anteriorly and bears two processes: ventral rostrum (*r*) and dorsal condylus (*c*) (Fig. 6A). The spicule is strongly curved; the posterior third of its blade is conical and forms an angle with its anterior part. The dorsal angular point is denoted as the spicule saddle (*sa*). The conical tip of the spicule bears a slightly widened extension called the cucullus (*cu*). A special rostral ligament (*rl*) joins the rostrum of each spicule to the ventral body wall (Figs. 6A; 7 D, E). The proctodeum lacks a gubernaculum, whose absence is the diagnostic feature of the fam. Aphelenchoididae.

The dorsal spicule protractor (*dsp*) is a bilateral pair of very thick longitudinal muscles that originate broadly from the *dBWM* muscles in the posterior portion of the tail and extend anteriorly toward the spicule. The contralateral muscles run together in close contact, but as they reach the spicule saddle, they diverge laterally and continue along the outer lateral sides of the spicules to insert on the spicular condylus (Figs. 5A–D; 7D–G).

The spicule saddle (*sa*) is an attachment point for two bilateral pairs of muscles. A pair of ventral spicule protractors (*vsp*) run from the saddle of each spicule posteroventrally to attach to the ventral body wall between the cloaca and the papillary tubercle (Figs. 5B–D; 7E, F). The second pair of muscles, the transverse saddle muscles (*tsm*), are attached both to the *vsp* and to the spicular saddle and in the retracted spicules extend laterally to anchor on the dorsolateral body wall (Figs. 5A, C, D; 7D–G).

6. Bursal muscles (*mb*).

The paired bursal muscles are continuations of the *cdl* and *pol* muscles; they are situated at the borders of the bursa, which is a leaf-like extension of the tail tip (Figs. 5A, B; 6A; 7A, B, D, F).

#### Chiloplacus sp.

(Figs. 5E–H, 8, 9).

**Figure 8:**
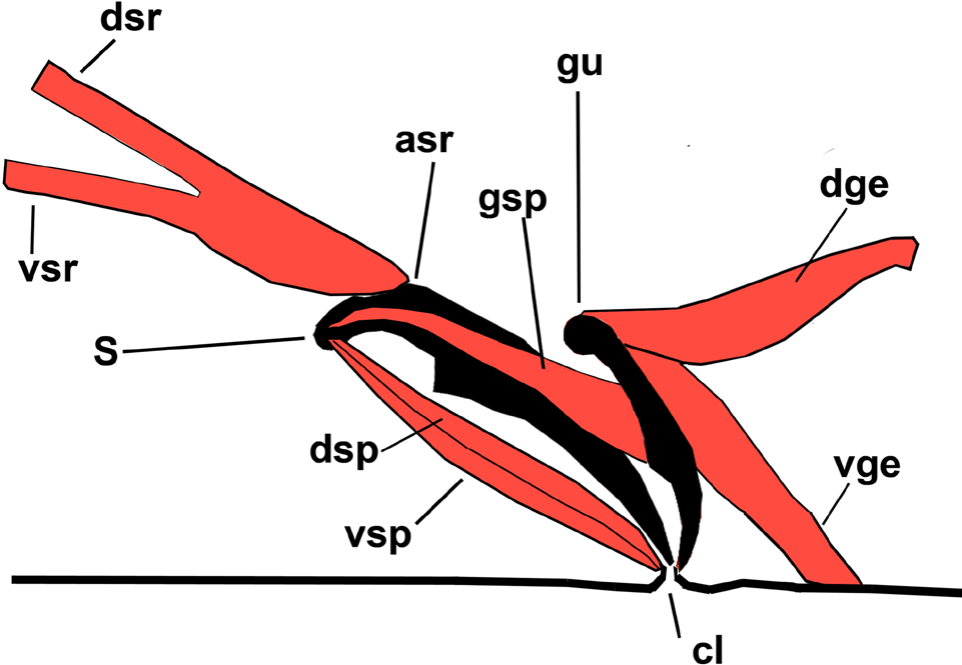
Schematic representation of spicular muscles of *Chiloplacus* sp. Abbreviations: asr—anterior spicule ridge; cl—cloacal opening; dge—dorsal gubernacular erector; dsp—dorsal spicule protractor; dsr—dorsal spicule retractor; gsp—gubernacular spicule protractor; gu—gubernaculum; s—spicule; vge—ventral gubernacular erector; vsp—ventral spicule protractor; vsr—ventral spicule retractor.

**Figure 9:**
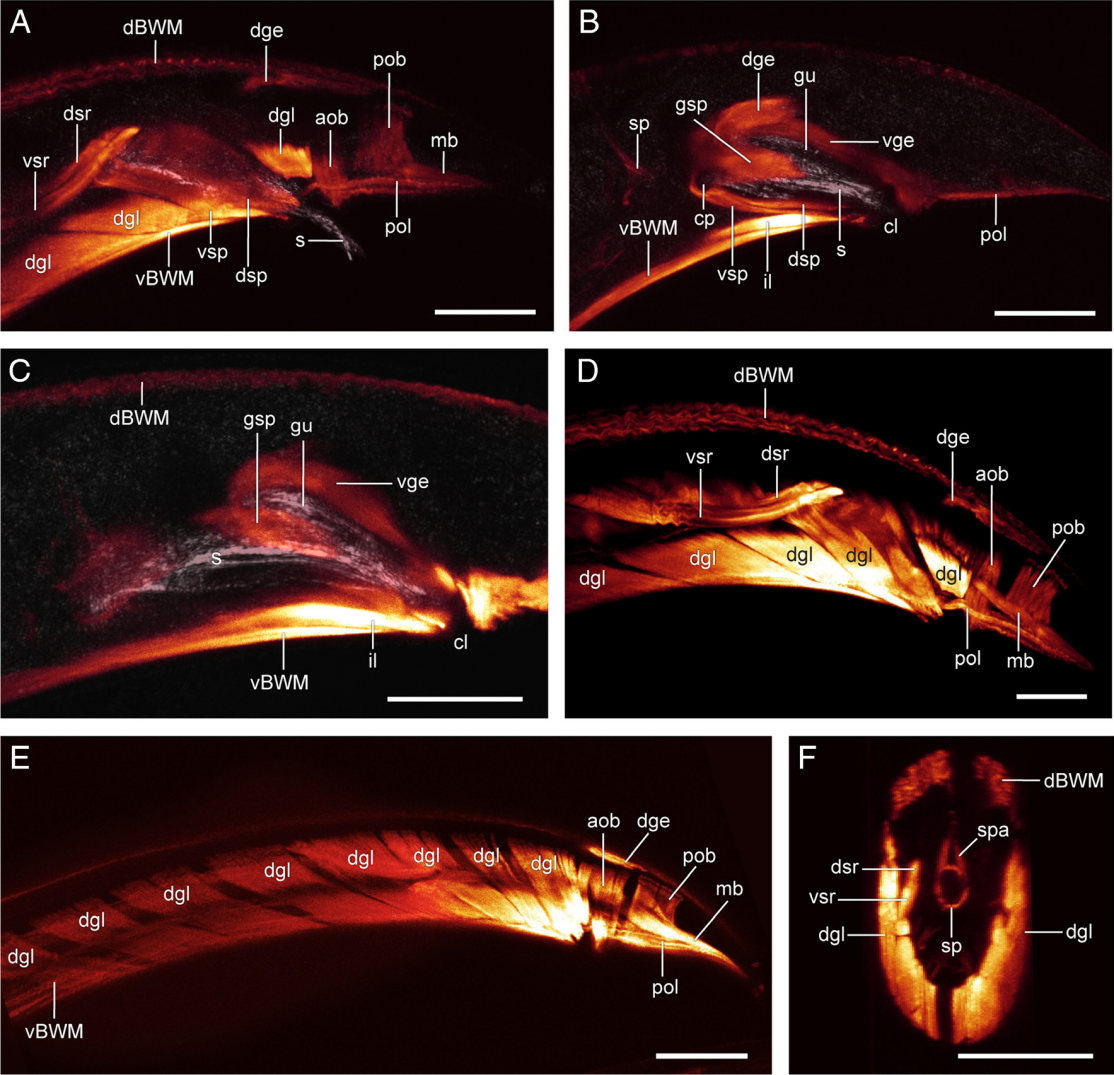
Posterior body region of males of *Chiloplacus* sp. showing muscles (orange) and spicules (s) with the gubernaculum (gu) in reflected light (grey). (A–D) Maximum projections of the tail in the *xy* plane showing spicular muscles at different depths within the body. (E) Maximum projection in the *xy* plane showing a row of eight diagonal muscles (dgl) on the right side of the body. (F) Maximum projection of the tail in the *yz* plane anterior to the spicules showing the sphincter muscle (sp) and its two dorsal arms (spa). Abbreviations: aob—anterior oblique muscle; cp—spicule capitulum; cl—cloacal opening; dBMW—dorsal body wall muscle; dge—dorsal gubernacular erector; dgl—diagonal muscle; dsp—dorsal spicule protractor; dsr—dorsal spicule retractor; gsp—gubernacular spicule protractor; gu—gubernaculum; il—inner longitudinal muscle; mb—bursa muscle; pob—posterior oblique muscle; pol—posterior outer longitudinal muscle; s—spicule; sp—sphincter muscle between mid-intestine and cloaca; spa—sphincter arms; vBMW—ventral body wall muscle; vge—ventral gubernacular erector; vsp—ventral spicule protractor; vsr—ventral spicule retractor. (Scale bars: A–D, F = 10 µm; E = 20 µm).

1. Body wall muscles (*BWM: dBWM and vBWM*).

The arrangement of the four body wall muscles is generally similar to that described for *B. mucronatus,* except that the *vBWM* are significantly longer than in the latter species and their posterior endings are located more anteriorly, at the lateral sides of the cloaca (Figs. 5E, F; 9A, C, E).

2. Longitudinal muscles (*il, cdl, pol*).

Although the overall arrangement of the longitudinal muscles is similar to that of *B. mucronatus,* there are some significant differences. The *pol* are much shorter than in *B. mucronatus*; they end anteriorly at the lateral sides of the cloaca and lie ventrally to the *mb* muscles, with which they appear to be in tight contact through much of their length (Figs. 5E, F; 9A, B, D, E). The *pm* muscles are lacking and only one inner longitudinal muscle (*il*) is present rather than two as in *B. mucronatus* (Figs. 5F; 9 B, C).

3. Precloacal copulatory diagonal muscles (*dgl*).

The *dgl* muscles are strap-shaped muscles arranged in a row of 8 on the right and 7 on the left side of the body (Figs. 5E, F; 9A, D–F). The *dgl* lie to the inside of the *vBWM* muscles; each muscle is oriented at about 45˚ angle to the vertical axis arising from the dorsolateral body wall near the lateral chords and ending on the ventrolateral body wall medial to the *vBWM* (Figs. 5E, F; 9A, D–F).

4. Postcloacal copulatory oblique muscles (*aob* and *pob*).

Two pairs of strap-shaped *ob* muscles, one anterior (*aob*) and one posterior (*pob*), are arranged in a row in the postcloacal region (Figs. 5E, F; 9A, D, E). Each muscle extends downward from the dorsolateral body wall to the ventral midline of the tail.

5. Spicule and spicule muscles.

The spicule is weakly C-shaped with a narrowly rounded capitulum, moderate handle and a curved blade (lamina) without an expanded tip appendage (Figs. 6B, C; 8; 9C).

Three sets of muscles are attached to the spicules: (i) one dorsal (*dsr*) and one ventral (*vsr*) pairs of spicule retractors; (ii) one ventral (*vsp*) and one dorsal (*dsp*) pairs of spicule protractors; and (iii) a pair of gubernacular spicule protractors (*gsp*).

Two pairs of spicule retractor muscles (*dsr* and *vsr*) arise from the anterior ridge (*asr*) of each spicule (Figs. 8; 9A, D) and continue anteriorly in close apposition to one another. About half the distance from their anterior endings, the *dsr* diverge slightly dorsally from the *vsr* and both muscles attach to the body wall at the lateral chord just dorsal to the *dgl* muscles (Figs. 5G, H; 8; 9A, D).

The ventral (*vsp*) and dorsal (*dsp*) spicule protractors remain in close contact throughout their length running anterodorsally from the lateral sides of the cloaca to insert on the capitulum of each spicule (Figs. 5G, H; 8; 9A, B). The gubernacular spicule protractors (*gsp*) arise posteriorly from the ventral surface of the proximal part of the gubernaculum and attach anteriorly to the spicule capitulum (Figs. 5F, H; 8; 9B, C).

6. Gubernaculum erectors (*dge* and *vge*).

The gubernaculum is a cuticular plate on the dorsal roof of the ventral part of the proctodeum (cloacal sac). It is an auxiliary structure that supports the spicules assisting in their protraction. Three pairs of muscles are attached to the gubernaculum: (i) dorsal (*dge*) and ventral (*vge*) pairs of gubernacular erectors and (ii) gubernacular spicule protractors (*gsp*; described above).

The dorsal gubernacular erectors (*dge*) originate from the dorsolateral body wall between the *dBWM* and *ob* and run anteroventrally to their insertion on the dorsal surface of the distal part of the gubernacular plate (Figs. 5G, H; 8; 9A, B, D, E). The ventral gubernacular erectors (*vge*) extend posteroventrally from the dorsal side of the gubernaculum to the posterior lip of the cloaca (Figs. 5G, H; 8; 9B, C). Both pairs of erectors appear to be attached to each other at their gubernacular insertion.

7. Bursal muscles (*mb*).

The bursa of *Chiloplacus* sp. is peloderan; its two alae surround the caudal region and the tail tip of the male. The *mb* are similar in morphology to those of *B. mucronatus* (Figs. 5E, F; 9A, D, E).

8. Sphincter muscle (*sp*) between the mid-intestine and cloaca.

The sphincter (*sp*) is a sexually dimorphic muscle consisting of a circular muscle ring encircling the intestine and a pair of dorsal processes (*spa*) that extend from the muscle ring and attach to the dorsal roof of the tail between the *dBWM* muscles (Fig. 9F).

## Discussion

Phalloidin staining for actin, in conjunction with confocal microscopy, is a powerful technique that provides detailed information on morphology and spatial arrangement of muscles. Its use in nematode research, however, has been limited, primarily due to the difficulty in rendering the cuticle sufficiently permeable for staining. This difficulty can be partially eliminated by using prolonged proteinase treatment, which is sufficient for adequate permeabilization of the cuticle, but does not incur excessive damage to the muscle tissue. Using this technique, the present study provided a detailed description of the vulval and male copulatory musculature in two phylogenetically distant nematode species: *Bursaphelenchus mucronatus* and *Chiloplacus* sp. The confocal microscopy studies of musculature in nematodes has so far been conducted only in *C. elegans* and our knowledge of morphology of copulatory and vulval musculature in other species derives exclusively from electron and light microscopy studies. Although only a few nematode taxa have so far been examined in detail, the results of these studies summarized in Table 1 may serve as a baseline for future studies on the diversity of reproductive musculature in nematodes. The following discussion provides comparison between the results of the previous research on vulval and male copulatory muscles (most notably, in *C. elegans*) and those obtained in the present study.

**Table 1.** List of Characters.

Class: Order: Family	Species or Genus or Family	C1	C2	C3	C4	C5	C6	C7	C8	C9	C10	C11	C12	C13	C14	C15	Reference
Enoplea: Dorylaimida: Actinolaimidae	*Actinca costata* (Schneider, 1935)	1	?	1	1	?	?	?	1	3	?	?	?	2	?	?	Coomans and Loof (1986)
Enoplea: Dorylaimida: Longidoridae	Longidoridae	1	3	1	1	1	2	2	1	3	1	1	?	2	?	?	Coomans (1975)
Enoplea: Enoplida: Capillariidae	*Capillaria hepatica* Bancroft, 1893	?	?	?	?	?	?	?	?	?	2	2	?	?	?	?	Wright (1978)
Enoplea: Isolaimida: Isolaimiidae	*Isolaimium* spp.	6	3	4	1	2	2	2	6	“1, 3	1	5	2	2	5	4	Timm (1969)
Enoplea: Mononchida: Mononchidae	*Hadronchus shakili* Ahmad (1980)	3	1	1	1	2	2	2	1	1	1	3	?	2	1	5	Ahmad (1980)
Enoplea: Trichocephalida: Trichuridae	*Trichuris muris* (Schrank, 1788)	?	?	?	?	?	?	?	?	?	1	2	?	?	?	?	Wright (1978)
Enoplea: Triplonchida: Trichodoridae	Trichodoridae	6	3	1	1	2	2	2	1, 2	6	1	2	?	2	?	?	Hooper (1975a)
Enoplea: Triplonchida: Trichodoridae	*Trichodorus*	6	3	1	1	2	2	2	1	6	1	2	?	2	?	?	Hooper (1975b)
Enoplea: Triplonchida: Trichodoridae	*Paratrichodorus*	6	3	1	1	2	2	2	2	6	1	2	?	1	?	?	Loof (1975)
Chromadorea: Plectida: Plectidae	*Neotylocephalus inflatus* (Yeates, 1967) Holovachov et al., 2003	?	?	3	?	?	?	?	?	?	?	?	?	2	?	?	Hussain (2006)
Chromadorea: Chromadorida: Cyatholaimidae	*Paracanthonchus caecus* (Bastian, 1865)	?	?	2	?	?	2	2	1	?	2	5	?	2	?	?	Vincx et al. (1982)
Chromadorea: Chromadorida: Cyatholaimidae	*Paracanthonchus heterodontus* (Schulz, 1932)	?	?	?	?	?	2	2	1	?	2	5	?	2	?	?	Vincx et al. (1982)
Chromadorea: Chromadorida: Cyatholaimidae	*Paracyatholaimus pugettensis* Wieser and Hopper, 1967	?	?	?	?	?	2	2	1	?	2	5	?	2	?	?	Zograf (2010)
Chromadorea: Monhysterida: Xyalidae	*Gonionchus africanus* Vincx and Furstenberg (1988)	?	?	?	?	?	?	?	1	?	2	?	1	2	2	1	Vincx and Furstenberg (1988)
Chromadorea: Monhysterida: Xyalidae	*Xyala psammonalis* Vincx and Furstenberg (1988)	?	?	?	?	?	?	?	1	?	2	?	1	2	2	1	Vincx and Furstenberg (1988)
Chromadorea: Monhysterida: Xyalidae	*Xyala striata* Cobb, 1920	?	?	?	?	?	?	?	1	?	2	?	1	2	2	1	Vincx and Furstenberg (1988)
Chromodorea: Rhabditida: Diplogastridae	*Acrostichus medius* Tahseen, Ahlawat, Asif and Mustaqim, 2016 (cited as *Acrostichus opimus* in Ahlawat, 2011)	?	?	?	1	?	?	?	3	4	3	?	?	?	2	1	Ahlawat (2011), Tahseen et al. (2016)
Chromodorea: Rhabditida: Diplogastridae	*Paroigolaimella helalii* (Tahseen et al., 2015) (cited as *Paroigolaimella dimorpha* in Ahlawat, 2011)	?	?	?	?	?	?	?	3	4	3	?	?	?	2	1	Ahlawat (2011), Tahseen et al. (2015)
Chromodorea: Rhabditida: Rhabditidae	*Caenorhabditis elegans* (Maupas, 1900)	4	?	2	1	2	2	2	2	4	3	4	0	1	3	3	Bird and Bird (1991), Lints and Hall (2009a, 2009b)
Chromodorea: Rhabditida: Rhabditidae	*Curviditis parilis* (Hussain, 2006)	?	?	2	?	?	?	?	2	4	3	4	?	1	?	?	Hussain (2006)
Chromodorea: Rhabditida: Rhabditidae	*Curviditis longicaudata* (Hussain, 2006)	?	?	2	?	?	?	?	2	4	3	4	?	1	?	?	Hussain (2006)
Chromodorea: Rhabditida: Rhabditidae	*Cuticularia macrodentata* (Hussain, 2006)	?	?	2	?	?	?	?	2	4	3	4	?	1	?	?	Hussain (2006)
Chromodorea: Rhabditida: Rhabditidae	*Pelodera scrofulata* Tahseen, 2014	4	?	?	1	?	?	?	2	4	3	4	?	1	?	?	Ahlawat (2011)
Chromodorea: Rhabditida: Rhabditidae	*Metarhabditis andrassyana* (Tahseen, Hussain, Tomar, Shan and Jairajpuri, 2004)	4	?	?	1	?	?	?	3	4	3	4	?	1	?	?	Ahlawat (2011)
Chromodorea: Rhabditida: Rhabditidae	*Metarhabditis costai* Asif, Prasad, Khan, Somasekhar and Tahseen, 2013 (cited as *M. distinctus* in Ahlawat, 2011)	4	?	?	1	?	?	?	3	4	3	4	?	1	?	?	Ahlawat (2011), Asif et al. (2013)
Chromodorea: Rhabditida: Rhabditidae	*Teratorhabditis andrassyi* (Tahseen and Jairajpuri, 1988)	7	2	3	4	2	2	2	?	?	?	?	?	?	?	?	Tahseen and Jairajpuri (1988), Tahseen et al. (2007)
Chromodorea: Rhabditida: Rhabditidae	*Teratorhabditis* synpapillata Sudhaus, 1985	7	2	3	4	2	2	2	?	?	?	5	?	?	?	?	Tahseen et al. (2007) (Fig. 1K)
Chromodorea: Rhabditida: Aphelenchoididae	*Ektaphelenchus obtusus* (Massey, 1956)	6	1	?	?	?	?	?	?	?	?	?	?	?	?	?	Bird and Bird (1991)
Chromodorea: Rhabditida: Hoplolaimidae	*Hoplolaimus pararobustus* (Schuurmans Stekhoven and Teunissen, 1938)	?	?	?	?	?	?	?	?	?	3	3	2	1	3	3	Coomans (1963)
Chromodorea: Rhabditida: Pratylenchidae	*Pratylenchus penetrans* (Cobb, 1917) Filipjev and Schuurmans Stekhoven, 1941	4	3	?	?	2	2	2	6	6	3	3	2	1	5	5	Wen and Chen (1976), Mai et al. (1977)
Chromodorea: Rhabditida: Pratylenchidae	*Hirschmanniella oryzae* (Van Breda de Haan, 1902) Luc and Goodey, 1964	4	1	?	?	?	?	?	5	5	3	3	1	1	4	2	Shakil and Jairajpuri (1976)
Chromodorea: Rhabditida: Cephalobidae	*Chiloplacus* sp.	5	3	4	4	2	1	2	2	4	4	5	2	1	5	4	This paper
Chromodorea: Rhabditida: Aphelenchoididae	*Bursaphelenchus mucronatus* Mamiya and Enda, 1979	6	1	4	4	1	1	1	4	2	5	6	2	1	5	5	This paper

Notes: Muscles of copulatory structures. C1–C15—characters; C1–C7—female, C8–C15—male. Character definitions: C1: Vulval dilators. 1: numerous fan-shaped muscles, not separated into bands; 2: numerous radial muscles, distinctly separated into bands; 3: 16 radial muscles: 8 anterior and 8 posterior, radiating from borders of round vulva; 4: 8 radial muscles: 4 pairs of vulval dilators; 5: 3 pairs of radial muscles; 6. 2 pairs of dilators; 7: absent or indistinct. C2: Vulval constrictors. 1: 4 muscles at margins of vulva (2 pairs); 2: thick rim of circular muscles around vulva; 3: constrictors absent, their functions performed by vaginal constrictors or vulval plate muscles. C3: Vaginal dilator (female). 1: fan-shaped radial layer not separated into bands; 2: numerous radial bands separated from each other; 3: 2 pairs of vaginal dilators at margins; 4: vaginal dilators absent, their functions performed by vulval dilators. C4: Vaginal constrictors. 1: ring muscle around vagina; 2: 2 pairs on each side of vagina; 3: 1 pair of oblique bands; 4: Absent, their functions performed by vulval constrictors. C5: Vaginal suspensors (radial bands preventing bulging out during egg laying). 1: present, fan-shaped broad muscle of posterior vulval lip (e.g. *su* in *Bursaphelenchus*); 2: absent. C6: Dilators of anterior vulval lip. 1: 1 pair of dilators (*fld* in *Bursaphelenchus*); 2: absent. C7: Constrictors of anterior vulval lip. 1: one pair of constrictors (*ta*); 2: absent. C8: Precloacal copulatory diagonal muscles. 1: Numerous, more than 8 pairs; 2: 7-8 pairs; 3: 4-6 pairs; 4. 2 pairs; 5: 1 pair; 6: absent or indistinct. C9: Postcloacal copulatory muscles. 1: numerous, separated from each other; 2: numerous amalgamated in one tissue-like layer, enveloping caudal region ventrally; 3: 4-6 pairs; 4: 2 pairs; 5: 1 pair; 6: absent or indistinct. C10: Spicule retractors. 1: 1 pair of muscles attached to proximal end of spicule extending anteriad to subdorsal or dorso-lateral body wall; 2: 2 pairs of muscles extending to dorso-lateral body wall; 3: 2 pairs of muscles: one pair attached to distal end of spicule extending anteriad to subdorsal body wall; another pair attached to capitulum and ventro-lateral body wall anteriorly; 4: 2 pairs starting as one band from spicule capitulum and then bifurcated anteriorly along lateral side with branches extending to latero-dorsal dorsal and latero-ventral body wall (Rhabditidae and Cephalobidae). C11: Spicule protractors. 1: protractor muscle attached to proximal end of the spicule, extending to subdorsal wall at tail tip; 2: spicule is enclosed by a capsule of suspensor muscles which act as protractor muscles; 3: 2 pairs of muscles enveloping spicule sheath from lateral, dorsal and ventral sides, anterior pair extending to body wall laterally and latero-ventrally at upper lip of cloaca, posterior pair extending from capitulum to latero-ventral body wall posterior to cloaca; 4: 2 pairs of muscles enveloping spicule from capitulum to its tip; dorsal pair extending to anal depressor and ventro-lateral body wall at posterior lip of cloaca; ventral pair extending from capitulum to ventro-lateral body wall at anterior lip of cloaca; 5: Three pairs of muscles: 2 pairs of anterior protractors running from capitulum to ventral body wall near anterior lip of cloaca and 1 pair of posterior protractor running caudally from capitulum to distal part of gubernaculum and then to latero-ventral body wall; 6: 2 pairs of muscles or the spicule capitulum ligaments: anterior dorsal pair extending from capitulum to subdorsal body wall and bifurcated at the spicule saddle; posterior ventral pair extending from spicule saddle to subventral body wall along tail; a pair of ligaments attached to capitulum rostrum and extending to subventral body wall; gubernaculum absent. C12: Rotator (a pair of muscles extending from middle part of spicule to ventral body wall). 1: present; 2: absent or indistinct. C13: Bursal muscles. 1: present; 2: absent or indistinct. C14: Gubernaculum retractors. 1: set of 4 muscles extending from lateral side of gubernaculum to dorsolateral body wall; 2: 1 pair of muscles extending from distal end of gubernaculum (apophysis) to the dorsolateral body wall; 3: 1 pair of muscles extending from proximal end of gubernaculum to dorso-lateral body wall; 4: 2 pairs: first pair are the sets of muscles from distal end of gubernaculum extending to dorsolateral body wall; second pair (seductors) extending from proximal end of gubernaculum to ventrolateral body wall; 5: Indistinct or absent. C15: Gubernaculum protractors. 1: 1 pair extending from proximal end of gubernaculum to ventrolateral body wall; 2: 2 pairs extending from proximal end of gubernaculum to ventro-lateral body wall; 3: 1 pair of muscles extending from distal end of gubernaculum to ventrolateral body wall; 4: 2 pairs of muscles extending from distal end of gubernaculum to ventro-lateral body wall (first pair) and to dorsolateral body wall (second pair); 5: Indistinct or absent. Symbol “?” means that data are not available.

The musculature of the vulval apparatus in the females of *Chiloplacus* sp. comprises radial muscles (dilators of the vulva) and muscles of the anterior vulval lip attached to the anterior inner vulval plate. The four pairs of radial dilators in *Chiloplacus* sp. are similar to the pattern in *C. elegans* and other Rhabditidae: *Pelodera scrofulata*, *Metarhabditis* spp., and *Hirschmanniella oryzae* (Table 1; Shakil and Jairajpuri, 1976; Bird and Bird, 1991; Ahlawat, 2011), but in *Chiloplacus* sp. the anterior pair of vulval dilators has been transformed into the muscles of the anterior vulval plate.

The musculature of the vulval apparatus in *Bursaphelenchus* is more complex than in *Chiloplacus* and includes longitudinal and diagonal muscles of the anterior and posterior lips of the vulva (*ma* and *dip*), longitudinal muscles of the external vulval flap (*flp*), transverse muscles of the vulval slit (*ta, ma* and *tp*), and posterior transverse muscle bands (*su*). The functional role of these muscles can be readily determined from their arrangement: longitudinal and diagonal muscles of the vulval flap and vulval lips (*ma*, *dip* and *flp*) are dilators serving to open the vulval slit structures upward and sideways, while the transverse muscles (*ta, ma* and *tp*) act as constrictors of the vulval slip and the *su* muscles may function as suspensors during mating and oviposition.

In *Bursaphelenchus,* the two opposing pairs of vulval dilators (*ma* and *dip*) show quadrilateral symmetry similar to that of the Rhabditidae; however, the anterior vulval flap has its unique paired *fld* dilators. Vulval constrictors of *B. mucronatus* (*ta* and *tp* pairs and an unpaired *mta* muscle at the slit-shaped vulval rim) have no identifiable counterparts in the other nematode species studied (Table 1); although two pairs of vulval constrictors are also present in *Hirschmanniella*, *Pratylenchus* (Pratylenchidae) and *Hadronchus* (Mononchidae) (Table 1; Shakil and Jairajpuri, 1976; Mai et al., 1977; Ahmad, 1980), they radiate outward from the rim of the vulval opening, rather than running along the rim as those of *B. mucronatus*.

The arrangement of muscles described here in the caudal body region of the males of *Chiloplacus* sp. is similar to that of *C. elegans* as reviewed by Lints and Hall (2009a, 2009b, Table 1). The significant differences concern the morphology of gubernacular muscles and there is also uncertainty about the identity of the anal depressor muscle. In *C. elegans*, the gubernacular muscles comprise two pairs attached to the dorsal body wall and functioning as erectors and retractors of the gubernaculum; *Chiloplacus* has a similarly arranged pair of erectors, but the second pair of muscles is attached to the body wall near the posterior cloacal lip also apparently acting as erectors rather than retractors. *Chiloplacus* sp. also has a third pair of gubernacular muscles (*gsp*) lacking in *C. elegans*. These muscles are connected to the spicules and their arrangement suggests that they may serve as additional spicule protractors pulling the spicular capitula toward the proximal part of the gubernaculum.

The anal depressor (*adp*) is a sexually dimorphic muscle that assists in defecation in the females but acts as an auxiliary spicule protractor in the males of *C. elegans* (Lints and Hall, 2009a, 2009b). In the males, its ventral contractile portion connects the gubernaculum and the posterior cloacal lip, while the non-contractile dorsal portion is H-shaped and is attached to the dorsal roof of the tail. No H-shaped muscle has been observed in the males of *Chiloplacus* sp., but the ventral gubernacular erectors (*vge*) of this species may be homologous to the ventral part of the anal depressor (*adp*) in *C. elegans*, as both have similar attachment to the gubernaculum and the posterior cloacal lip. Since the dorsal H-shaped part of the *adp* lacks contractile elements in *C. elegans*, it cannot be visualized by phalloidin staining and it is likely that in *Chiloplacus* sp. this part of the muscle is also present but cannot be detected by the methods used in the present study.

The sphincter (*sp*) muscle of both *Chiloplacus* sp. and *C. elegans* appears to be modified in the males to assist in insemination by closing the mid-intestine and drawing it dorsally from the vas deferens thus expanding the cloaca for sperm passage (Lints and Hall, 2009b). The sphincter of *Chiloplacus* sp., however, has two dorsal arms, while only one arm persists in the males of *C. elegans* (in the juveniles the sphincter has four arms).

The copulatory muscle system of *Bursaphelenchus* differs significantly from those of *Chiloplacus* sp. and *C. elegans*. Like the other Aphelenchoididae, *Bursaphelenchus* lacks a gubernaculum and has strongly curved spicules. Only three pairs of muscles are associated with the spicules: two pairs of spicule protractors (the dorsal pair inserted on the condylus and the ventral pair attached to the spicular saddle), and the third pair of transverse muscles (*tsm*) extending from the saddle laterally. No apparent retractor muscles have been identified.

Although in *B. mucronatus* the gubernaculum is lacking, the arrangement of muscles attached to the spicular saddle bears certain resemblance to that of the gubernacular muscles in *Chiloplacus* sp. and *C. elegans*. The *vsp* muscles of *B. mucronatus*, like the *vge* muscles of *Chiloplacus* and the *adp* muscles of *C. elegans*, are attached to the posterior cloacal lips and have a similar orientation; the *tsm* muscles of *B. mucronatus* are positioned essentially like the gubernacular erectors in *Chiloplacus* and *C. elegans* except that their origin on the body wall is located more ventrally than in the two latter species. This indicates a possible homology between the conical endings of the spicules of *B. mucronatus* and the gubernaculum of *Chiloplacus* and *Caenorhabditis*. It is possible that the gubernaculum has become fused with the anterior body of the spicule in the ancestral line of the Aphelenchoididae and the gubernacular muscles have transformed into the musculature of the spicular saddle.

In *Chiloplacus* sp., the female and the male are oriented parallel to each other during mating (Fig. 1 G) and the male embraces the vulval region of the female with the caudal bursal alae. When the spicules are inserted into the female’s vulval opening, the gubernaculum serves as a support for the spicular tips, as it was shown for *C. elegans* (Lints and Hall, 2009a, 2009b) and *Pelodera strongyloides* (Wagner and Seitz, 1981); in *Chiloplacus* sp. it probably also assists in spicule protraction serving as attachment for the gubernacular spicule protractors lacking in *C. elegans*. It is unlikely that in *Chiloplacus* sp. the ventral gubernacular erector (presumably homologous to the anal depressor) functions as an auxiliary spicule protractor as does the anal depressor in *C. elegans* (Lints and Hall, 2009b), because in the latter species the contractile part of the anal depressor is aligned and coupled with that of the spicule protractors, while in *Chiloplacus* sp. these muscles are positioned at a distance from each other. Spicule protraction in *Chiloplacus* sp. is therefore accomplished by three muscles: two pairs of spicule protractors and one pair of gubernacular spicule protractors with the gubernaculum raised by two pairs of gubernacular erectors, and retraction is performed by two pairs of spicule retractors inserted on the anterior spicular ridges.

In *Bursaphelenchus*, the mates are oriented perpendicular to each other during copulation; the male clasps the female vulval region by coiling its tail around the female’s body (Figs. 1E, F; 2; Vieira et al., 2009). The bursa of *Bursaphelenchus* is different from the male bursa of *Chiloplacus* and other Rhabditidae. It is a terminal flap-like appendage bordered by glandular papillae, its morphology being specifically adapted for holding the female during mating in perpendicular orientation (Ryss et al., 2005, 2021). Spicule protraction in *B. mucronatus* is apparently accomplished by two muscles: the dorsal and ventral protractors (the latter presumably being homologous to the anal depressor as discussed above). The ventral protractors may also function as spicule rotators similar to the muscles attached to the mid-portion of the spicules in the family Xyalidae (order Monhysterida) and in *Hirschmanniella oryzae* (Paratylenchidae) (Table 1: Character 12; Shakil and Jairajpuri, 1976; Vincx and Furstenberg, 1988). It is not clear how spicule retraction is achieved as no apparent retractors have been described in *B. mucronatus*, but it is possible that spicules are retracted by a combined action of the transverse saddle muscles (*tsm*) and the *rl* ligaments attached to the spicular rostra. The rostral ligaments are evidently stretched and tensed during spicule protraction and it may be assumed that once the spicule protractors are relaxed the ligaments would be able to pull the spicules back into the cloaca by elastic recoil. The *tsm* are oriented transversally in retracted spicules and may pull the spicules apart, but they assume a diagonal orientation when the spicules are extruded so that the force vector of these muscles is directed into the cloaca and their contraction may therefore assist in spicule retraction.

Differences between *Bursaphelenchus* and *Chiloplacus* in mating arrangement are reflected in different patterns of the outer muscles of the tail. In *B. mucronatus*, the caudal oblique muscles (*ob*) are a pair of broad transverse muscle sheets consisting of numerous parallel myofibers, while in *C. elegans* and *Chiloplacus* there are two distinct pairs of muscles (*aob* and *pob*). A broad bilateral muscle sheet may represent the basal condition; during mating the contraction of these muscles causes the tail to curl into a ring enabling the male to grip tightly the female’s vulval region (Figs. 1 E, F; 2). The long rows of *ob* muscles have also been reported in *Spironoura chelydrae* (Harwood, 1932) (order Ascaridida: family Kathlaniidae) and *Hadronchus shakili* (order Mononchida: family Mononchidae) (Mackin, 1936; Ahmad, 1980; Table 1). In the *C. elegans* and *Chiloplacus* males, a short postcloacal zone of two pairs of oblique muscles may be explained by the peloderan shape of their bursae provided with paired caudal alae. The male uses the alae to clasp the female’s vulval region embracing it from the sides (Fig. 1G) and the oblique muscles come into play only during the ‘turning phase’ of mating (Lints and Hall, 2009a). It is likely that when the peloderan bursa has appeared in evolution, the zone of postcloacal ventral muscles has been reduced from a long muscle sheet (as in *Bursaphelenchus* and Mononchidae) to only two pairs (*C. elegans* and *Chiloplacus*). In *B. mucronatus*, the terminal bursa is a small sticky appendage with a pair of *mb* muscles.

Another adaptation to the perpendicular mating orientation in *Bursaphelenchus* is the vulval flap with developed *fld* dilators and vulval slit constrictors (paired *ta* and *tp* and the unpaired *mta* muscle). This unique vulval flap-and-slit muscle system wedges the inserted endings of the spicules in place during mating functioning effectively as a lock for the spicules. The terminal bursa and broad oblique muscle sheets that cause the tail to coil during mating together with strongly curved spicules act as a “key” designed for this type of vulva. Such mutual evolutionary adjustment of copulatory organs in both sexes exemplifies the “key and lock” principle in morphology of copulatory structures (Ryss et al., 2005; Behr, 2008; Vieira et al., 2009; Ryss and Subbotin, 2017).

In the females of *Chiloplacus* sp., the rounded vulva is not covered on the outside by an external vulval flap; the vulva is opened by contraction of radial dilators (Figs. 3B; 4E) and the spicules in the males are shaped as almost straight cones (Figs. 6B, C; 8). During mating, the function of the “key” is performed by spicules and the bursal alae with their ventrolateral longitudinal constrictors (*cdl*, *mb* and *pol* muscles). Such configuration may be described as a “key without a lock”; only male copulatory structures are responsible for the connection of partners during mating and insemination. Similar mating has been described for *C. elegans* (Lints and Hall, 2009a, 2009b). Wagner and Seitz (1981) pointed out that the male bursa of *Pelodera strongyloides* serves as a mould for the adhesive secretion of the cloacal glands; this ensures fixation of the spicule tips opposite the vulval opening of the female.

The comparison of the copulatory musculature between the species examined in the present paper and those studied previously allows for some preliminary phylogenetic conclusions. If the *gsp* in *Chiloplacus* is indeed homologous to the anal depressor, the spicular muscle systems of *C. elegans* and *Chiloplacus* can be regarded as essentially similar differing only in the gubernacular retractors (absent in *Chiloplacus*) and gubernacular spicule protractors (absent in *C. elegans*). The gubernacular retractors may have been lost in the ancestral lineage of *Chiloplacus* because of the changes in the role played by the anal depressor (=*gsp*), while the gubernacular spicule protractors could have become reduced in the *Caenorhabditis* lineage, because the anal depressor took over the function of an auxiliary spicule protractor. The remaining spicular muscles may have been present in the common ancestral stock of both groups, i.e. a group that includes clades 11 (“Cephalobomorpha”) and 9A (“Rhabditomorpha”) in the total nematode phylogeny of Megen et al. (2009). In the phylogeny of Megen et al. (2009), clade 10B that includes *Bursaphelenchus* spp. and other Aphelenchoididae is more basal than clades 11 and 9A, and this clade appears to exhibit a combination of ancestral and derived traits: a broad postcloacal muscle sheet is likely to be a primitive condition, while the system of spicular muscles has been modified as a result of reduction of the gubernaculum.
